# Longitudinal Change and Tracking of Health-Related Physical Fitness in Adolescents Aged 12–16 Years: A Structured Narrative Review

**DOI:** 10.3390/children13091208

**Published:** 2026-09-07

**Authors:** Souhail Bchini, Dhouha Moussaoui, Abedelmalek Kalefh Tabnjh, Anissa Bouassida, Nadhir Hammami, Fatma Hilal Yagin

**Affiliations:** 1 Research Unit (UR22JS01) “Sport Sciences, Health and Movement”, High Institute of Sport and Physical Education of Kef, University of Jendouba, Kef 7100, Tunisia; bouassida_anissa@yahoo.fr (A.B.); nadhir.hammami@issepkef.u-jendouba.tn (N.H.); 2 Higher Institute of Sport and Physical Education of Ksar Said, University of La Manouba, Manouba 2010, Tunisia; douhamoussaoui@yahoo.fr; 3 Department of Cariology, Institute of Odontology, The Sahlgrenska Academy, University of Gothenburg, 40530 Gothenburg, Sweden; 4 Department of Applied Dental Sciences, Faculty of Applied Medical Sciences, Jordan University of Science and Technology, Irbid 22110, Jordan; 5 Department of Pathology, Saveetha Medical College and Hospital, Saveetha Institute of Medical and Technical Sciences, Chennai 602105, Tamil Nadu, India; 6 Department of Biostatistics, Faculty of Medicine, Malatya Turgut Ozal University, Malatya 44210, Türkiye; hilal.yagin@gmail.com

**Keywords:** physical fitness, adolescent health, cardiorespiratory endurance, muscular strength, mental well-being, public health

## Abstract

**Highlights:**

**What are the main findings?**

- Cardiorespiratory fitness peaks in early adolescence before plateauing or declining around age 15 in a single Icelandic cohort, while muscular strength and power show significant sex differences consistent with androgen-mediated muscle accrual in boys, although this mechanism was not directly assessed in the reviewed studies.
- Health-related physical fitness is increasingly stable during adolescence, with an individual’s overall fitness rank showing its strongest reported stability, in one cohort of adolescent girls, between the ages of 15 and 17.

**What are the implications of the main findings?**

- Ages 12 to 13 may represent a favorable window for school-based fitness interventions to improve fitness levels, especially for disadvantaged adolescents, before fitness rank becomes increasingly stable—though this review did not evaluate intervention efficacy directly.
- Given that adolescent fitness rank is highly stable and declining adolescent fitness may persist into adulthood with associated health risks, implementing universal school-based fitness monitoring is a plausible public health priority.

**Abstract:**

**Background/Objectives**: The period between 12 and 16 years of age coincides with peak pubertal change, declining habitual physical activity, and the consolidation of lifelong behavioral patterns, making it a critical window for understanding how fitness develops and how stable (or unstable) it is over time. This review aims to synthesize longitudinal evidence addressing how HRPF changes and tracks in adolescents aged approximately 12 to 16 years, the sex- and maturation-related modifiers of these patterns, the methodological approaches used to assess them, and the secular and behavioral context within which they occur. **Methods**: A structured narrative review was conducted searching four electronic databases—Google Scholar, PubMed, Web of Science, and Scopus—using terms combining concepts of adolescence, health-related physical fitness, and longitudinal study design published up to April 2026. Reference lists of retrieved systematic reviews and meta-analyses were snowball-searched. Eligible studies employed longitudinal or repeated-measures designs in samples overlapping the 12–16-year age range; no formal risk-of-bias appraisal or quantitative pooling was undertaken, consistent with the narrative-review design. Findings were synthesized narratively by HRPF component and thematic domain. **Results**: Cardiorespiratory fitness rises through early adolescence before plateauing or declining from approximately age 15 in one longitudinal cohort, more markedly in boys; muscular power and strength diverge increasingly by sex, a pattern consistent with androgen-mediated gains in lean mass in boys, although this mechanism was not directly tested here; flexibility remains the most stable component; and body composition acts as both an outcome and a determinant of other fitness test performance. Within-adolescence tracking is moderate-to-high for most components in the single available 3-year cohort of adolescent girls that reported component-specific tracking coefficients (e.g., lower-limb power β = 0.98, aerobic endurance β = 0.75, trunk muscular endurance β = 0.51), and substantially stronger than tracking from childhood into adolescence. Secular trends indicate population-level fitness has declined in many countries over recent decades, with adolescents showing greater decline than children. **Conclusions**: Early-to-mid adolescence appears to be a critical and time-limited opportunity to consider for fitness-promoting intervention: fitness rank is still somewhat malleable, but shows progressively stronger tracking with age—a pattern of rank stability that should not itself be read as evidence about responsiveness to intervention. Methodological heterogeneity in test batteries, follow-up duration, and tracking statistics limits direct comparability across studies. Future research should incorporate maturity-status indicators, multi-wave designs, and harmonized assessment protocols.

## 1. Introduction

Physical fitness is widely regarded as one of the most powerful biomarkers of health available in pediatric and adolescent populations, summarizing the combined influence of habitual physical activity, body composition, genetic endowment, and biological maturation into a small set of measurable physiological capacities. Health-related physical fitness (HRPF) is generally distinguished from skill- or performance-related fitness by its direct relevance to morbidity and functional health, and it is conventionally subdivided into cardiorespiratory endurance, muscular strength and endurance, flexibility, and body composition [1]. A substantial body of evidence has linked higher HRPF in youth to a more favorable cardio metabolic risk profile, lower adiposity, better bone health, and improved psychological wellbeing [1], while also predicting cardiovascular and metabolic outcomes—and, in some cohorts, premature mortality—decades later in adulthood [2,3].

Adolescence, and in particular the years between approximately 12 and 16, represents a uniquely consequential developmental window for fitness. This period overlaps with the adolescent growth spurt and the most rapid phase of pubertal maturation, during which lean mass, stature, and cardiopulmonary capacity expand non-linearly and asynchronously between the sexes [4,5]. At the same time, longitudinal evidence consistently shows that habitual physical activity declines through adolescence, a decline that is steeper in girls than boys and that has been linked to changes in autonomy, body image, and the displacement of active play by academic and social demands [6,7]. The convergence of rapid biological change and declining physical activity makes this age range a plausible “critical period” in which fitness trajectories that persist into adulthood may be established.

Two related but distinct questions arise from this developmental context. The first concerns change: how do the various components of HRPF develop, on average, across the early-to-mid adolescent years, and do trajectories differ by sex, component, or maturity status? The second concerns tracking: to what extent do adolescents who are fit (or unfit) relative to their peers at one time point remain so at a later time point? Tracking—typically defined as the maintenance of relative rank within a group over time, or the ability to predict later values from earlier ones [8]—has direct public health relevance, because strong tracking would imply that fitness status in early-to-mid adolescence is a reliable indicator of future fitness, supporting early identification and intervention, whereas weak tracking would imply more fluidity and a wider window for change. Tracking describes the stability of relative rank; it does not, by itself, indicate whether a given trajectory is more or less responsive to intervention, a distinction we return to throughout this review.

The 12–16-year window is mechanistically distinct from the broader childhood-to-adulthood span in at least four ways that compound one another. First, it uniquely straddles the peak height velocity (PHV) inflection point in both sexes—around age 12 in girls and age 14 in boys—meaning that the two sexes are, on average, at qualitatively different stages of pubertal development during the same chronological-age band [9,10]. Second, this window coincides with the steepest documented decline in habitual physical activity, a decline that is both more pronounced than in childhood and more directly linked to the health-risk behavior consolidation that tracks into adulthood [6,7]. Third, as the tracking evidence reviewed in Section 5 demonstrates, fitness rank transitions from low-to-moderate stability during the childhood-to-adolescence span to moderate-to-high stability within adolescence itself—with the highest tracking statistics of all observed precisely across the 15–17-year interval that closes this window, albeit in cohorts whose sampled ages extend to, or slightly beyond, the upper edge of the 12–16 range that is this review’s primary focus [4,8]. Fourth, from an intervention perspective, this is the period in which school-based physical education reaches its greatest contact hours but is also most threatened by competing academic demands, making it an especially high-leverage but time-limited opportunity to act. For all these reasons, evidence synthesized specifically for this age band provides distinct practical value over reviews that treat adolescence as a uniform developmental block.

Despite a sizeable empirical literature, our search did not identify a recent structured narrative synthesis focused specifically on the 12–16-year age band—a claim based on the search strategy described in Section 2 rather than on a separate, systematic novelty check, no recent structured narrative synthesis has focused specifically on the 12–16-year age band, which spans the period of greatest pubertal flux and is therefore mechanistically distinct from broader child-to-adult tracking studies. The purpose of this structured narrative review is to synthesize longitudinal evidence addressing how HRPF changes and tracks in adolescents aged approximately 12 to 16 years, the sex- and maturation-related modifiers of these patterns, the methodological approaches used to assess them, and the secular and behavioral context within which they occur.

## 2. Methods

### 2.1. Review Approach

This is a structured narrative review. The aim was to provide a broad, integrative synthesis of longitudinal evidence on HRPF change and tracking in adolescents, organized around a pre-specified thematic framework, rather than to exhaustively catalogue every eligible study or to pool effect sizes, as would be expected of a systematic review or meta-analysis. The structured approach differs from a purely narrative review in that the search strategy, eligibility criteria, and thematic synthesis framework were defined a priori (see Section 2.2, Section 2.3 and Section 2.4); it differs from a systematic review in that it does not employ formal risk-of-bias appraisal or statistical pooling. This design was considered appropriate given the heterogeneity of fitness components, test batteries, follow-up intervals, and tracking statistics used across the literature, which limits direct quantitative synthesis, while the pre-specified structure ensures transparency and replicability of the synthesis process. No formal quality-weighting procedure was applied to the retrieved literature; single-cohort primary studies (e.g., refs. [4,8,11]) are therefore reported throughout as illustrative of the available evidence rather than as generalizable population estimates, and are explicitly distinguished in the text from pooled meta-analytic sources (e.g., refs. [12,13,14,15]), which carry greater evidentiary weight.

### 2.2. Search Strategy

A literature search was conducted across four databases: Google Scholar, PubMed, Web of Science, and Scopus. Search terms combined concepts relating to the population, exposure/outcome, and design, including combinations of: adolescent, youth, and teenager; health-related physical fitness, physical fitness, cardiorespiratory fitness, muscular fitness, muscular strength, flexibility, body composition; and longitudinal, tracking, trajectory, follow-up, secular trend, cohort study. Reference lists of retrieved systematic reviews and meta-analyses were hand-searched to identify additional primary studies (a snowballing approach), and forward citation searching was used to locate more recent studies citing key methodological or conceptual papers.

### 2.3. Eligibility Considerations

Studies were included if they met all of the following criteria: (a) the sampled or measurement window overlapped the approximate 12–16-year age range (studies with broader ranges, e.g., 6–18 or 7–17 years, were included where the 12–16 window fell within the sampled range, and are flagged in-text where a specific finding derives predominantly from ages outside 12–16); (b) the design was longitudinal or repeated-measures (cohort, panel, or tracking study), or the study was a systematic review/meta-analysis synthesizing such designs; and (c) the study reported on at least one recognized component of health-related physical fitness (cardiorespiratory endurance, muscular strength/power/endurance, flexibility, or body composition), a quantitative tracking or change statistic, or a secular-trend estimate meeting these same population and design criteria. Cross-sectional secular-trend studies were additionally included where they provided population-level context for longitudinal findings, and are labeled as cross-sectional rather than longitudinal throughout the text and in Table 1. Studies were excluded if they: sampled exclusively early childhood (under 6 years) or exclusively adult cohorts; addressed skill-related fitness components (e.g., balance, coordination) in isolation without a health-related fitness component; or reported no extractable quantitative fitness, tracking, or secular-trend outcome.

### 2.4. Synthesis

Findings were organized thematically around (1) definitions and assessment of HRPF, (2) developmental change by fitness component, (3) tracking/stability of fitness over time, (4) sex and maturational modifiers, (5) secular trends, and (6) determinants of change. This structure was chosen to reflect the dual focus of the review—change and tracking—while situating both within the broader biological and behavioral context of adolescence.

### 2.5. Summary of Key Studies

Table 1 presents the subset of retrieved studies that reported a quantitative tracking, change, or secular-trend statistic, or a longitudinal health-outcome association, meeting the eligibility criteria in Section 2.3, together with study characteristics, follow-up duration or assessment window, baseline assessment battery, and outcome measures for the longitudinal, tracking, and large-scale surveillance studies most central to this review. Studies are organized by thematic group: (1) developmental change and tracking, (2) sex and maturation, (3) secular trends and surveillance, and (4) health outcomes. Full bibliographic details are given in the numbered reference list.

The combined search across the four databases identified 1340 records; after removal of duplicates, 812 unique records remained for title/abstract screening; 715 were excluded at this stage as not meeting the population, design, or outcome criteria in Section 2.3, leaving 97 records assessed at full text, of which 50 were excluded, principally for lacking an extractable longitudinal tracking, change, or secular-trend statistic. A total of 47 studies met all eligibility criteria and were retained for synthesis, of which 27 are summarized in Table 1.

## 3. Defining and Assessing Health-Related Physical Fitness

HRPF is conventionally operationalized through a multi-component test battery rather than a single measure. The ALPHA (Assessing Levels of Physical Activity) health-related fitness test battery, developed through a series of systematic reviews of predictive validity, criterion validity, and reliability together with field studies in schools, selected the 20 m shuttle run test for cardiorespiratory fitness, handgrip strength and the standing broad jump for musculoskeletal fitness, and body mass index, skinfold thickness, and waist circumference for body composition [30,31]. The test battery was shown to be valid, reliable, feasible, and safe for assessing HRPF in children and adolescents for population-level health monitoring, and the full protocol can be administered to a class-sized group by a single physical education teacher in under two hours [30]. Cardiorespiratory fitness in particular has been argued to merit dedicated, ongoing population surveillance alongside other established health indicators, given its strong and consistent association with current and future health outcomes in youth [32], a rationale distinct from, and not directly evidenced by, the musculoskeletal-fitness criterion standards reported in [33]; the previous sentence linking [33] to “ROC-based aerobic-capacity standards” has been removed pending verification of the correct source. Related batteries in widespread use include Eurofit and FITNESSGRAM, each emphasizing broadly similar constructs (aerobic capacity, muscular strength/endurance, flexibility, and body composition) but differing in specific test protocols, which complicates direct comparison of absolute scores across studies and countries [34].

These three batteries remain the most widely implemented internationally and share a common emphasis on tests that are inexpensive, require minimal equipment, and can be delivered by classroom or physical education staff rather than specialist exercise physiologists [34]. Eurofit, developed in the 1980s and subsequently adopted across the majority of European countries and beyond, has itself accumulated a large normative base: pooled analysis of more than 2.7 million Eurofit performances from 9- to 17-year-olds across 30 European countries was used to derive sex- and age-specific normative values, providing one of the most extensive reference datasets available for any youth fitness battery [35]. A review of the test–retest reliability of the individual Eurofit items concluded that the plate-tapping, handgrip strength, sit-ups, bent-arm hang, 10 × 5 m agility shuttle run, and 20 m multistage shuttle run tests show moderate-to-excellent reliability, that the sit-and-reach and standing broad jump tests show good-to-excellent reliability, and that the flamingo balance test shows only moderate-to-good reliability [36], supporting the inclusion of the strength, endurance, and jump items—but cautioning more strongly against the balance item—in longitudinal designs where measurement stability over repeated administrations is essential for distinguishing true developmental change from measurement noise.

FITNESSGRAM, the test battery most widely used in North American school-based surveillance, similarly assesses aerobic capacity (commonly via the PACER shuttle run), muscular strength and endurance (e.g., curl-up and push-up tests), flexibility (e.g., sit-and-reach), and body composition, and has likewise demonstrated acceptable reliability and validity for use with adolescent populations when administered by trained staff [37]. Because ALPHA, Eurofit, and FITNESSGRAM differ in the specific protocols used for ostensibly equivalent constructs—for example, in cardiorespiratory fitness testing via the 20 m shuttle run versus the PACER, which use different audio cadences and stage progressions—raw scores are not directly interchangeable across studies that used different batteries, and tracking or secular-trend analyses that pool across batteries typically require conversion to standardized (z-score or percentile) units rather than comparison of absolute values [34].

The conceptual rationale for prioritizing HRPF over skill-related fitness rests on its demonstrated associations with health outcomes. An influential narrative review summarizing the relationship between fitness and health in young people concluded that cardiorespiratory fitness is associated with total and abdominal adiposity, that both cardiorespiratory and muscular fitness are associated with established and emerging cardiovascular disease risk factors, that improvements in muscular fitness and speed/agility (rather than cardiorespiratory fitness specifically) appear to benefit skeletal health, and that cardiorespiratory fitness gains are linked to improvements in depression, anxiety, mood, self-esteem, and academic performance [1]. This breadth of association is part of the justification for treating HRPF, rather than any single component, as the outcome of interest in longitudinal and tracking research.

Beyond the choice of test battery, a further assessment consideration specific to the 12–16-year age range is whether fitness is expressed in absolute terms, relative to body mass, or relative to fat-free (lean) mass. Because body mass and its composition change substantially and asynchronously between the sexes across this period, the choice of denominator can materially alter both the apparent developmental trajectory and the magnitude of sex differences observed at a given age [4]—absolute and mass-relative fitness values are not interchangeable expressions of the same trajectory but distinct outcomes that can diverge in direction, as shown directly in Section 4.1, a methodological issue revisited throughout this review (Section 4.1 and Section 10) wherever absolute and relative fitness trajectories diverge.

Table 2 summarizes the component tests, typical equipment requirements, and key assessment notes for the three test batteries most frequently used to assess HRPF in adolescents aged 12–16 years.

## 4. Longitudinal Change in HRPF Components Across Adolescence

### 4.1. Cardiorespiratory Fitness

Cardiorespiratory fitness (CRF) follows a broadly curvilinear trajectory across childhood and adolescence, rising through childhood and early adolescence before slowing or plateauing in mid-to-late adolescence. Because absolute and lean-mass-relative CRF are distinct, non-interchangeable outcomes (Section 3), they are reported separately below. In a ten-year longitudinal study of a single cohort of Icelandic youth followed from age 7 to 17, maximal power output, both in absolute terms and relative to lean mass, increased for both boys and girls up to age 15, after which absolute power plateaued for both sexes, while power relative to lean mass plateaued in girls but declined in boys between ages 15 and 17 [4]. Boys showed higher absolute power from age 15 and higher relative power from age 9 onward, illustrating a widening sex gap that emerges well before the 12–16-year window closes [4]. The same study explicitly identified age 15 as a critical transition point, with CRF values beginning to plateau in girls and decline in boys from that age, a finding from this single cohort that reinforces the plausibility, but does not by itself establish generalizability, of the 12–16 window as the period in which this inflection occurs [4].

### 4.2. Muscular Strength, Power, and Endurance

Musculoskeletal fitness—encompassing maximal strength, explosive power, and muscular endurance—shows the most pronounced sex divergence of any HRPF component during early-to-mid adolescence, a pattern consistent with, though not directly tested against, the markedly greater androgen-related gains in lean mass experienced by boys. Musculoskeletal fitness reflects the ability of a muscle or muscle group to exert force maximally, explosively, or continuously without fatigue, alongside the ability to move a joint through its full range of motion [19], and longitudinal data across this age band typically show boys pulling progressively further ahead of girls in absolute strength and power measures as adolescence progresses, even as relative (mass-adjusted) measures sometimes show a narrower gap [16].

### 4.3. Flexibility and Body Composition

Flexibility tends to be the most stable HRPF component across adolescence in absolute terms, while body composition—typically indexed via BMI, skinfolds, fat mass, or fat-free mass—shows the clearest unfavorable secular shift across recent decades (see Section 8), independent of within-individual developmental change. Longitudinal analysis of adolescent girls found that fat mass was inversely associated with standing broad jump, sit-ups in 60 s, and squats in 60 s, while positive associations were observed between fat mass and both the 800 m and 400 m run times; fat-free mass showed the opposite pattern, being positively associated with broad jump, sit-ups, and squats, and negatively associated with run times [11], illustrating how body composition functions as a determinant of, rather than merely a component alongside, other fitness measures during this developmental period [11,38].

### 4.4. Composite Picture

Taken together, the available longitudinal data depict the 12–16-year window as a period of continued, but decelerating and increasingly sex-divergent, improvement in most HRPF components, with cardiorespiratory fitness in particular showing signs of plateau or decline by mid-adolescence—earlier in girls than in boys for some indices, and earlier in boys for others, depending on whether absolute or mass-relative values are examined [4,11,38].

Table 3 summarizes the principal developmental pattern reported for each HRPF component across the 12–16-year window, by sex, as synthesized from the longitudinal studies discussed in Section 4.1, Section 4.2 and Section 4.3.

## 5. Tracking and Stability of HRPF During Adolescence

### 5.1. Magnitude of Tracking Within Adolescence Versus Childhood-to-Adulthood

A key distinction in the tracking literature is between tracking within adolescence (e.g., age 12 to age 16) and tracking from childhood or adolescence into adulthood, which typically spans a much longer interval and is subject to greater attenuation. A systematic review and meta-analysis of tracking from childhood and/or adolescence to adulthood found that cardiorespiratory fitness, muscular strength, and muscular endurance show moderate stability, independent of the specific test used, with this moderate tracking being slightly higher in women than men and higher when tracked from adolescence rather than from childhood; flexibility showed the highest stability of the components examined [12]. This pattern—stronger tracking from adolescence than from childhood, and a flexibility advantage in stability—has direct implications for the 12–16-year window: because adolescent fitness tracks more strongly into adulthood than childhood fitness does, this age range may represent a more reliable point at which to identify at-risk individuals than earlier childhood measurements [12].

Within adolescence itself, tracking coefficients tend to be markedly higher than across the full childhood-to-adulthood span. In a three-year follow-up of a single cohort of adolescent girls measured at ages 15 and 17 (an age range at, or beyond, the upper edge of this review’s 12–16 focus), the highest tracking coefficient was observed for explosive power of the lower extremities (β = 0.98), followed by flexibility (β = 0.89), body composition (β = 0.88), speed endurance (β = 0.86), aerobic endurance (β = 0.75), muscular endurance of the lower extremities (β = 0.65), and muscular endurance of the trunk (β = 0.51), with tertile ratings (high/moderate/low fitness groups) remaining largely stable across the two time points [8]. The authors concluded that moderate-to-high tracking of physical fitness in adolescent girls suggests that interventions aiming to increase fitness should probably begin in early adolescence, a conclusion about intervention timing that follows from the rank-stability pattern observed but was not itself tested through an intervention in this or other reviewed studies [8].

A complementary cardiorespiratory-fitness-specific analysis reinforces this contrast between developmental phases. Across the childhood-to-adolescence transition, in the single Icelandic cohort described in Section 4.1, statistically significant odds ratios for remaining in the same CRF category were observed between ages 9 and 15 and between ages 9 and 17, but not between ages 7 and 15 or ages 7 and 17, while the highest odds ratios of all were observed within adolescence itself, between ages 15 and 17, for boys, girls, and the combined sample alike, and higher odds ratios were generally observed for boys than for girls across age comparisons [4]. This pattern—weak tracking across the childhood–adolescence boundary but strong tracking within adolescence—suggests that early-to-mid adolescence functions as a period during which fitness rank stabilizes and becomes considerably more predictive of future status than it was earlier in development.

### 5.2. Tracking of a Broader Battery Across the School-Age-to-Adolescence Transition

Evidence extending slightly earlier, from childhood into adolescence, situates the 12–16-year window within a longer trajectory. A longitudinal study examining nine fitness measures—including flexed arm hang, jump and reach, standing long jump, agility shuttle run, dash speed, endurance shuttle run, and sit-and-reach—at five age points from 6 to 18 years found that boys outperformed girls on fitness measures at most time points, and that children who started in the lowest fitness tertile had a markedly poor chance of moving into the high-fitness tertile over developmental time, whereas children in the middle tertile had a meaningfully better chance of moving up [16]. This pattern suggested that achieving at least an average level of fitness in childhood improves the likelihood of becoming highly fit, or remaining at least averagely fit, later in development, and that health- and performance-related fitness remain amenable to change through carefully designed intervention, particularly for those not already in the lowest tertile [16]. This finding nuances the tracking narrative: while rank-order stability is generally moderate-to-high, the probability of upward mobility is asymmetric, with the lowest-fitness group facing the greatest difficulty escaping that status—a pattern with direct relevance for targeting intervention resources during the 12–16-year window.

### 5.3. Interpreting Tracking Coefficients

Tracking is most often quantified using Spearman or Pearson correlations between time points, tracking (stability) coefficients derived from mixed or generalized estimating equation models, or categorical approaches such as the percentage of individuals remaining within the same tertile or quartile across measurement waves, sometimes supplemented by odds ratios for remaining in a given category. Tracking has been defined in the literature in two complementary ways: as a tendency of individuals to maintain their rank within a group over a period of time, and as the ability to predict future observations based on earlier values [8]. Tracking coefficients describe rank stability and should not be interpreted as evidence that fitness trajectories are resistant to intervention, a distinction that was not directly assessed by any study reviewed here and that we make explicit before turning, in Section 11, to the implications of this tracking evidence for intervention timing. Because different studies report different statistics (correlation coefficients, beta/tracking coefficients, odds ratios, or percentage agreement), direct numerical comparison across studies should be made cautiously; nonetheless, the qualitative pattern—moderate-to-high tracking within adolescence, particularly for power, flexibility, and body composition, somewhat lower tracking for muscular endurance measures—recurs across multiple independent cohorts [8,12,16].

Table 4 collects the tracking statistics discussed in Section 5.1 and Section 5.2, grouped by component and by the age span over which tracking was assessed, to make the contrast between within-adolescence and childhood-to-adulthood tracking, and the relative ordering of components, easier to compare at a glance.

## 6. Sex Differences and the Role of Biological Maturation

Sex differences in both the level and the trajectory of HRPF widen substantially across the 12–16-year window, reflecting divergent pubertal timing and tempo. Boys generally show steeper gains in absolute cardiorespiratory and muscular fitness, a pattern consistent with, though not a direct test of, greater testosterone-driven increases in lean mass and cardiac/pulmonary dimensions; this mechanism is drawn from the broader endocrinology and auxology literature and was not itself measured within the observational fitness studies synthesized here, while girls’ trajectories are more strongly shaped by increases in fat mass that can partially offset gains in relative (mass-adjusted) fitness [4,11]. Inter-individual and between-sex differences in puberty timing are associated with biological changes—including increased height, weight, strength, and fat mass—and psychosocial changes such as shifts in self-esteem and body image concerns, both of which influence fitness-related behavior during adolescence [5]. Earlier-maturing boys may benefit from height, weight, and strength advantages that facilitate continued activity participation, whereas earlier-maturing girls are typically less active, a pattern attributed to changes in body composition, increased self-consciousness, and lower self-concept [5].

The biological basis of this divergence lies in the timing and tempo of the adolescent growth spurt itself. On average, girls reach peak height velocity (PHV) at around 12 years of age, gaining roughly 9 cm per year at the peak and approximately 25 cm in total height over the pubertal growth period, with the spurt typically coinciding with Tanner breast stage 3 [9]. Boys, by contrast, reach PHV roughly two years later, around age 14, with a higher peak velocity of approximately 10–10.3 cm per year and a larger total height gain of around 28 cm, typically coinciding with Tanner genital stage 3–4 [9,39]. Because the 12–16-year window captures girls at or just past PHV but boys approaching or at PHV, the two sexes are, on average, at materially different points of their respective growth spurts during the same chronological-age band—a mismatch that is a primary driver of the widening fitness gap documented in Section 4 [10]. This timing difference is compounded by sequencing: in boys, the peak gains in height, weight, and muscle mass occur together, whereas in girls these peaks occur in a more staggered sequence, and strength and power gains in both sexes typically follow, rather than coincide with, PHV itself [10], meaning that the fitness consequences of the growth spurt lag somewhat behind the most visible anthropometric changes.

Because chronological age is therefore an imperfect proxy for developmental stage, especially in this rapidly changing window, the maturity-offset method—which estimates an individual’s age relative to their own PHV using anthropometric predictors—has become a standard tool for separating maturation-driven from purely age-related change in youth fitness research [17], with refinements to the original prediction equations subsequently developed to improve accuracy across a wider range of body types [18]. These prediction equations carry non-trivial individual-level errors even after refinement, a limitation discussed further in Section 10. Studies that classify adolescents by maturity status rather than chronological age consistently report that early-maturing boys hold a performance advantage in strength- and power-based tests over their later-maturing peers of the same age, while comparable maturity-based advantages in girls are less consistently observed and are sometimes offset by the unfavorable association between earlier maturation and higher fat mass [10].

Maturation also appears to interact with the behavioral side of the fitness equation—physical activity—in ways that are not fully resolved in the literature. Evidence is mixed regarding whether adolescent declines in physical activity are closely tied to biological maturation: some work concludes that sex-related differences in biological maturity contribute to sex-related differences in physical activity during adolescence, while other longitudinal work in adolescent girls found that neither maturation nor absolute changes in physical size directly influenced physical activity change, leading reviewers to describe the influence of sex, age, and maturation on physical activity change during adolescence as equivocal overall [40]. This uncertainty is methodologically important: studies that index development purely by chronological age, without accounting for maturity status (e.g., age at peak height velocity or pubertal staging), may misattribute maturation-driven change to age-related or purely behavioral mechanisms, or vice versa. Reviewers have recommended that future longitudinal studies incorporate biological maturation markers alongside chronological age specifically to help clarify how much of the observed decline in activity (and, by extension, fitness) is due to developmental timing rather than simply getting older [6].

The tracking literature reflects similarly modest but consistent sex differences. As noted above, tracking from childhood/adolescence to adulthood has been found to be slightly higher in women than men [12,41], even though boys tend to show higher odds ratios for remaining in the same CRF category across most age comparisons within adolescence [4]. These apparently divergent findings likely reflect differences in outcome (rank stability vs. absolute level), time span (within-adolescence vs. into-adulthood), and fitness component examined, and underscore the need for studies to report sex-stratified results explicitly rather than presenting only pooled estimates. They also reinforce the broader point that sex and maturation are conceptually distinct but empirically entangled moderators: two adolescents of the same chronological sex and age may nonetheless be at very different points of biological maturity, and studies that fail to disentangle the two risk attributing to “sex” differences that are more properly attributable to differential maturity timing between the average boy and average girl at a given age [10].

## 7. Determinants and Correlates of Change: Physical Activity, Body Composition, and Socioeconomic Context

### 7.1. The Role of Declining Physical Activity

Because cardiorespiratory and muscular fitness are partly trainable, the well-documented adolescent decline in habitual physical activity is among the most plausible behavioral drivers of fitness trajectory change across the 12–16-year window. Longitudinal studies have shown that physical activity declines, on average, from adolescence to young adulthood, although population-level averages mask substantial individual variation, with some subgroups of adolescents increasing or maintaining activity while others decrease it [7]. Identifying these distinct trajectories has practical importance, since determinants of change or maintenance of (in)activity may differ between subgroups and represent valuable information for targeted health promotion [7], implying that fitness-promoting interventions in this age band may need to be tailored to different activity-trajectory subgroups rather than applied uniformly.

The decline in activity does not appear uniform across activity domains. A systematic review of longitudinal changes in physical activity domains found that organized physical activity tended to increase during childhood and either increase or remain stable through the transition into adolescence, while non-organized activity tended to remain stable among girls but decline among boys during adolescence, and active transport showed an increase during childhood followed by a plateau or decline in adolescence [42]. This domain-specific pattern suggests that the aggregate “decline in physical activity” often cited in the fitness literature may obscure more nuanced shifts—for example, a move from unstructured to structured activity—that could differentially affect specific HRPF components (e.g., organized sport may preserve muscular power and CRF even as overall incidental activity declines).

### 7.2. Body Composition as Both Outcome and Determinant

As outlined in Section 4.3, body composition operates bidirectional in relation to other fitness components: it is itself a tracked HRPF outcome, while simultaneously acting as a determinant of performance on strength, power, and endurance tests. This bidirectional relationship has been demonstrated longitudinally, with body composition changes (fat mass and fat-free mass) predicting subsequent performance on jump, sit-up, squat, and endurance-run tests in adolescent girls followed over three years [11] (Section 4.3), which addresses cardiorespiratory fitness and later health risk rather than this body-composition tracking finding. Clinical intervention data extend this picture to adolescents with overweight/obesity specifically, where a thirteen-month multidisciplinary lifestyle intervention study in adolescents aged 13–16 examined which combination of socioeconomic factors, early-life factors, body composition change, fitness change, and physical activity change best predicted longitudinal change in cardio metabolic risk profile [43], reflecting the broader principle that fitness, adiposity, and physical activity changes are best understood as an interdependent system during this developmental window rather than as independent processes.

### 7.3. Socioeconomic and Contextual Factors

Beyond individual biological and behavioral determinants, broader contextual disruptions illustrate how sensitive adolescent fitness trajectories are to environmental change. A one-year longitudinal study of Portuguese adolescents in grades 5 through 12, assessing body composition, aerobic fitness, speed, agility, strength, and flexibility before the COVID-19 pandemic, after the initial lockdown, and two months after in-person classes resumed, found that body composition (waist circumference) and aerobic fitness (maximal oxygen uptake) deteriorated after the lockdown but improved again once in-person classes resumed [24]. This natural experiment demonstrates the responsiveness of adolescent HRPF—particularly its aerobic and body-composition components—to relatively short-term changes in opportunity for physical activity, reinforcing that observed “tracking” reflects not pure biological determinism but a continuous interplay between underlying trajectory and proximal environmental context.

Socioeconomic status (SES) is an important but frequently underreported moderator of both baseline HRPF level and the rate of fitness decline across adolescence. Lower-SES adolescents tend to have lower baseline cardiorespiratory fitness, less access to organized sport and safe recreational infrastructure, and higher rates of obesogenic behavior, all of which compound across the 12–16-year window to widen fitness disparities that are already present at school entry [7,16]. The asymmetric upward-mobility pattern identified in the tracking literature—wherein adolescents in the lowest fitness tertile face markedly poor odds of reaching the high-fitness tertile over developmental time (Section 5.2)—is likely to be concentrated disproportionately among lower-SES adolescents, since the same structural barriers that depress initial fitness also reduce the probability of trajectory improvement [16]. Gender interacts with SES in ways that are not fully characterized in the available longitudinal literature: girls from lower-SES backgrounds face compounded disadvantages from both the sex-specific activity-decline pattern (Section 6) and reduced access to structured sport. School-based universal fitness monitoring—such as the ALPHA battery or Eurofit, which require no individual financial outlay from students—may therefore be particularly valuable for identifying at-risk adolescents who would not self-select into voluntary fitness programmers or clinical screening, making equitable access to PE-based surveillance a public health priority aligned with the goals of this Special Issue [30,32].

## 8. Secular Trends: The Population-Level Backdrop to Individual Tracking

Individual-level longitudinal change and tracking should be interpreted against a backdrop of declining population-level fitness in many countries, which complicates comparisons of absolute fitness values across cohorts measured in different eras. A systematic review analyzing secular trends in cardiorespiratory endurance, relative muscle strength, muscle power proxies, and speed in children and adolescents aged 6–18, drawing on 24 studies from 16 countries with a combined sample exceeding 860,000 children and adolescents, found that the majority of studies reported a decline in physical fitness over time, with performance in endurance, strength, and flexibility decreasing, while trends in speed and coordination were inconsistent across studies [14]. Earlier work cited within that review had found a significant decline of approximately 10% in fitness between 1975 and 2000, most pronounced for endurance and flexibility, with subsequent analysis showing that the decline in fitness was smaller for children than for adolescents [15]—a finding directly relevant to the present review’s focus, since it implies the adolescent years carry a disproportionate share of population-level fitness erosion.

The magnitude of decline specific to cardiorespiratory fitness has been quantified directly in large pooled analyses. A study spanning 19 high-income and upper-middle-income countries found that cardiorespiratory fitness in children and adolescents declined by a sample-weighted 7.3% between 1981 and 2014, a moderate but consistent decline observed across most, though not all, of the included countries [13]. This 7.3% figure and the ~10% figure reported above are not directly comparable: the former reflects cardiorespiratory fitness specifically across 1981–2014 in 19 countries [13], while the latter reflects a composite of fitness components across the earlier, partly overlapping 1975–2000 window in a different set of studies [15]; the difference in magnitude therefore reflects differing scope and time period rather than conflicting evidence. A complementary analysis of secular trends in pediatric aerobic fitness test performance globally described this decline as precipitous from around 1970 onward, a pattern that the more recent update covering data to 2015 suggests has since slowed, with the negative trend in cardiorespiratory endurance appearing to have reached something closer to a floor in recent years even as absolute levels remain below their historic peak [15]. Other fitness components central to this review show comparable, if more recently documented, secular patterns: pooled analyses of standing broad jump performance and of sit-ups performance, each spanning several decades and millions of children and adolescents internationally, have similarly been used to track explosive power and muscular endurance trends over time, complementing the cardiorespiratory-fitness-focused literature and reinforcing that the secular decline documented in this section is not confined to aerobic capacity alone [15].

More recent national surveillance data reinforce this picture while adding nuance about component-specific and temporal variation. The published protocol for a decennial Slovenian national monitoring programme spanning five decades reports, on the basis of the programme’s accumulated surveillance data, component-specific secular patterns that differ by fitness measure: the most notable decline has occurred in cardiorespiratory fitness, although this trend appears to have stabilized for some groups after approximately 2012; muscular strength, after a prolonged decline, has shown modest recent improvement; muscular power continues to show a slight negative trend; and speed performance has improved slightly to moderately since 2002, though only in specific subgroups). As these figures are drawn from the programme’s protocol paper rather than a dedicated results publication, they should be treated as a description of the programme’s reported surveillance patterns rather than as independently peer-reviewed effect estimates; substantive results publications from this programme, once available, should be cited alongside or in place of the protocol paper. These trends have unfolded in parallel with a tripling of the global age-standardized prevalence of childhood obesity between 1990 and 2021 [23], a contextual association consistent with the cross-sectional and longitudinal body-composition findings discussed in Section 4.3 and Section 7.2. A separate repeated cross-sectional national surveillance programme monitoring Croatian children and adolescents aged 11–14 between 1999 and 2014 similarly reported declines in health-related fitness over the period, broadly consistent with the Slovenian and pooled international evidence and suggesting that the secular pattern documented across high-income European countries is not confined to any single national surveillance system [22].

Trends are not uniform globally. Large-scale Chinese national survey data spanning more than three decades illustrate how secular patterns can reverse direction across different sub-periods: an analysis of eight successive national surveys of Chinese adolescents aged 13–18 between 1985 and 2019 found that mean body height, weight, and BMI increased significantly over the 34-year period [19], while a parallel analysis of rural Chinese youth found that fitness improved between 1985 and 2000 (with the exception of flexibility), declined across most components between 2000 and 2010, and showed mixed, component- and sex-specific rebounds and continued declines between 2010 and 2019 [20]. Similarly, in Brazil, repeated cross-sectional surveillance from 2005 to 2022 covering more than 65,000 children and adolescents provided evidence that fitness is declining for the majority of youth, with this decline diverging asymmetrically and becoming more extreme in more recent years, even as a “fit minority” did not show the same decline [21]. These divergent regional and sub-period trends indicate that any cohort-specific tracking or change estimate must be interpreted in light of the secular era in which the underlying data were collected, since the same chronological age band (12–16 years) may carry systematically different absolute fitness levels depending on testing decade and national context.

A further, more recent disruption to secular trend lines has been the COVID-19 pandemic and its associated school closures and activity restrictions. A systematic review and meta-analyses of European data covering the period during and after the pandemic found that several HRPF components weakened across the pandemic period relative to pre-pandemic trajectories, broadly corroborating the natural-experiment evidence from Portugal already discussed in Section 7.3 [25], and underlining that recent-era secular trend estimates for the 12–16-year age band may carry a pandemic-related discontinuity that should be considered separately from longer-run, pre-pandemic secular patterns when comparing cohorts tested before versus after 2020.

Table 5 consolidates the secular-trend studies discussed in this section by region, study period, sample size, and principal direction of change, to make the cross-national heterogeneity described above easier to compare at a glance.

## 9. Health Implications of Fitness Trajectories Across Adolescence

The practical significance of documenting change and tracking in this age band derives from the well-established links between youth fitness and later health. Findings across the literature show that greater physical fitness—primarily cardiorespiratory endurance and muscular fitness—is associated with reduced all-cause mortality and a lower risk of developing a wide range of non-communicable diseases in adulthood, independent of potential confounders, and that acquiring adequate fitness levels from an early age is related to better body composition and a healthier cardio metabolic profile later in life [12]. A systematic review of longitudinal studies focused specifically on cardiorespiratory fitness reported that higher CRF in childhood and adolescence predicts a more favorable cardiovascular risk-factor profile in later adolescence and adulthood, including more favorable blood pressure, lipid, and adiposity trajectories [26], while earlier longitudinal evidence linking childhood-to-adulthood fitness decline to adult obesity and insulin resistance suggests that the same processes that erode fitness tracking (Section 5) may simultaneously erode metabolic health, reinforcing that fitness decline across this period should not be viewed merely as a performance outcome but as a plausible early marker of cardio metabolic trajectory [27]. Because tracking analyses (Section 5) indicate that fitness rank achieved by mid-adolescence is substantially more predictive of later fitness than fitness measured in childhood, the 12–16-year window can reasonably be framed as a key leverage point: interventions delivered here are acting on a trajectory that is both increasingly stable in rank (and therefore, plausibly, harder to shift through brief intervention, though this was not directly tested) more proximally predictive of adult fitness and health than interventions delivered earlier in childhood.

Self-perceived health appears to track alongside objectively measured fitness across adolescence. A combined cross-sectional and longitudinal study of children and adolescents found that higher HRPF was associated with better self-rated health, with the association evident both in single time-point comparisons and over follow-up, suggesting that the health correlates of fitness documented through objective clinical and biochemical markers are also reflected in how adolescents themselves perceive their health and wellbeing [44]. At the other end of the wellbeing spectrum, fitness has also been examined in relation to psychological constructs beyond depression and anxiety specifically: a study within the EHDLA cohort examined associations between physical fitness, experiential avoidance, and psychological inflexibility in adolescents, reporting that lower fitness was associated with less adaptive patterns on these constructs [45], extending the health-relevance of HRPF beyond the cardio metabolic and mental-health-symptom outcomes already discussed (above and in the paragraph below) into broader dimensions of psychological functioning that may be particularly salient during the identity-formation tasks of early-to-mid adolescence.

Muscular fitness specifically has accumulated a substantial and partly independent evidence base. A systematic review and meta-analysis of longitudinal studies concluded that higher muscular fitness in childhood and adolescence is associated with a more favorable body composition, lower cardio metabolic risk, and better bone health later in life, with associations that are generally independent of cardiorespiratory fitness [3], while a further systematic review focused specifically on muscular fitness and cardio metabolic variables in children and adolescents reported consistent inverse associations between muscular fitness and clustered cardio metabolic risk scores, blood pressure, and adiposity markers across the available longitudinal and cross-sectional evidence [29]. Taken together with the cardiorespiratory-fitness evidence above, this supports treating both major HRPF components—rather than cardiorespiratory fitness alone—as independently informative targets for monitoring and intervention across the 12–16-year window.

Beyond physical health outcomes, an increasingly substantial body of evidence links HRPF, and physical activity more broadly, to adolescent mental health. A systematic review and meta-analysis synthesizing the relationship between physical fitness and mental health in youth found cardiorespiratory and muscular fitness to be associated with fewer depressive and anxiety symptoms and better self-esteem, although effect sizes were generally small and the strength of evidence varied across mental health outcomes and fitness components [28]. A related systematic review and meta-analysis of physical activity and sedentary behavior in preschoolers, children, and adolescents similarly concluded that physical activity is favorably associated with indicators of mental health, including depression, anxiety, and self-esteem, while higher sedentary behavior was generally associated with poorer mental health indicators [46]. Because physical activity is both a determinant of HRPF (Section 7.1) and independently associated with mental health, these findings suggest that fitness-promoting intervention in the 12–16-year window may carry mental health co-benefits alongside the physical health and tracking-related rationale already discussed, although the mental health evidence to date is more strongly established for physical activity as a behavior than for fitness as a physiological outcome.

A particularly important implication of the tracking evidence (Section 5) for mental health and motivation concerns the self-reinforcing nature of fitness rank in adolescence. Adolescents who are consistently low-fitness relative to peers are not only at higher cardio metabolic risk but are also more likely to develop a lower physical self-concept—a negative perception of their own physical competence and appearance—which in turn reduces motivation to engage in physical activity, thereby further depressing fitness. This bidirectional cycle between objective fitness status and psychological self-concept has been reported in cross-sectional adolescent samples [45,47], which cannot establish the direction of this relationship, and has important practical consequences: it means that fitness-promoting interventions in this age band should not target physical capacity alone, but should simultaneously address motivational and self-efficacy barriers, particularly for adolescents in the lowest fitness tertile who face the greatest risk of remaining trapped in a low-fitness trajectory. Approaches that emphasize mastery, competence, and intrinsic enjoyment over performance comparison—consistent with autonomy-supportive physical education pedagogy—may therefore be especially well-suited to the 12–16-year window, when physical self-concept is being actively constructed and is most responsive to positive experience.

This dual character—strengthening rank stability alongside continued, if narrowing, plasticity (Section 5.2)—is consistent with the broader conclusion drawn across multiple primary studies reviewed here, namely that moderate-to-high tracking of fitness in the adolescent cohort reviewed in Section 5.1 implies that interventions aiming to raise fitness levels might reasonably begin in early adolescence, before tracking strengthens further—a hypothesis motivated by, but not directly tested through, the tracking evidence reviewed here [8].

## 10. Methodological Considerations and Limitations of the Evidence Base

Several methodological issues recur across the reviewed literature and constrain the strength of conclusions that can be drawn.

Heterogeneity of test batteries and outcome metrics. Studies vary in their choice of field tests (e.g., 20 m shuttle run vs. cycle ergometer for cardiorespiratory fitness; handgrip vs. standing broad jump vs. sit-ups for muscular fitness), and in whether fitness is expressed in absolute terms or relative to body mass or lean mass—a distinction shown to materially affect both developmental trajectories and sex comparisons (Section 4.1) [4,16]. This heterogeneity limits the comparability of tracking coefficients and change estimates across studies.

Variability in tracking statistics. As noted in Section 5.3, tracking has been quantified using Spearman/Pearson correlations, regression-derived tracking (beta) coefficients, percentage agreement across tertiles/quartiles, and odds ratios for category maintenance [4,8,12]. These statistics are not numerically interchangeable, and narrative (rather than purely quantitative) synthesis is therefore necessary when integrating findings across studies, as undertaken in this review.

Follow-up duration and number of waves. Many studies in this literature involve only two measurement waves (e.g., baseline and a two- or three-year follow-up) [8,19], which can characterize change and pairwise tracking but cannot describe the shape of the developmental trajectory (e.g., whether change is linear, accelerating, or decelerating across the 12–16-year span) as precisely as designs with three or more waves [4,16].

Attrition and sample representativeness. Longitudinal designs are inherently vulnerable to differential attrition, where less fit, less active, or lower-SES adolescents may be disproportionately lost to follow-up, potentially biasing tracking coefficients toward the experience of more advantaged or more compliant participants. Collecting longitudinal data carries acknowledged limitations in terms of cost, time, and drop-out rates [8], a constraint that likely contributes to the relatively short follow-up intervals and modest sample sizes characteristic of much of this literature.

Confounding of age and maturation. As discussed in Section 6, studies that rely solely on chronological age without incorporating maturity status (such as age at peak height velocity or pubertal staging) risk conflating maturation-driven and purely chronological-age-driven change, a limitation explicitly acknowledged in recent reviews calling for greater use of maturity-offset methods in future longitudinal designs [6,40]. Maturity-offset and predicted-PHV methods themselves carry non-trivial individual-level prediction error even after refinement [18]; studies relying on predicted rather than directly observed PHV should therefore be interpreted with this additional measurement uncertainty in mind.

Secular confounding of cross-cohort comparisons. As shown in Section 8, the same age band can show systematically different absolute fitness levels depending on the decade in which it was sampled [14,15,20,21]; comparing tracking or change estimates derived from cohorts tested in different eras, without accounting for secular trend, risks misattributing era-driven differences to developmental ones.

Table 6 summarizes these six recurring methodological limitations alongside their principal consequence for interpreting change and tracking estimates, and the mitigation most commonly recommended in the literature reviewed.

## 11. Implications for Intervention Timing and Setting

The longitudinal change and tracking evidence synthesized in this review carries indirect implications for the design, timing, and delivery of fitness-promoting interventions during adolescence; no intervention-efficacy trials were systematically reviewed here, and the implications below should be read as hypotheses generated by the tracking and change evidence rather than as evaluated recommendations. Three settings are considered here: school-based physical education, community and clinical programmers, and emerging digital approaches.

### 11.1. School-Based Physical Education

School-based physical education represents the most universal and equitable lever for population-level fitness in this age band, reaching adolescents regardless of family income, organized sport participation, or individual motivation. The tracking data synthesized in Section 5 carry a specific message for PE: because within-adolescence fitness rank shows progressively stronger stability across the 12–16-year window, the marginal effectiveness of a given PE-based intervention may plausibly be greatest in early adolescence (ages 12–13), before tracking strengthens and the structural barriers to upward tertile mobility accumulate—a hypothesis consistent with, but not directly established by, the tracking evidence reviewed here. PE programmers that prioritize increases in moderate-to-vigorous-intensity activity volume, and that explicitly target cardiorespiratory and muscular fitness rather than skill-based outcomes alone, are most likely to shift fitness trajectories in ways that persist beyond the intervention period [30,32]. The ALPHA and Eurofit batteries reviewed in Section 3 are feasible for class-sized delivery, making population-level fitness surveillance within PE settings a practical reality rather than a research aspiration.

### 11.2. Community and Clinical Settings

The asymmetric mobility finding (Section 5.2)—that adolescents in the lowest fitness tertile face markedly poor odds of moving into higher tertiles over developmental time—provides a rationale for targeted, individually tailored intervention for this high-risk subgroup, though this review did not evaluate whether such intervention is in fact effective. Community and clinical settings (youth health services, pediatric obesity clinics, school nurse programmers) are well-placed to deliver such interventions, particularly for lower-SES adolescents who may not access them through organized sport (Section 7.3). The cardio metabolic evidence reviewed in Section 9—linking adolescent fitness decline to adult obesity, insulin resistance, and cardiovascular risk—supports a preventive framing: identifying low-fitness adolescents at age 12–13 and delivering multidisciplinary lifestyle support at this stage is likely more cost-effective than clinical management of cardio metabolic disease in adulthood [27,43], given the asymmetric mobility pattern described in Section 5.2; this rationale concerns the timing and cost-effectiveness of intervention and should not be read as implying that adolescents with more stable fitness rank are less responsive to intervention. In clinical settings, the SES-stratified nature of fitness disparities (Section 7.3) implies that screening tools should be applied universally rather than selectively, to avoid systematic under-identification of at-risk adolescents from disadvantaged backgrounds.

### 11.3. Digital and Emerging Approaches

The methodological limitations identified in Section 10—particularly the heterogeneity of assessment batteries, the short follow-up intervals of many studies, and the difficulty of capturing fitness trajectories with sufficient temporal resolution—suggest that emerging digital health technologies offer both a practical surveillance tool and a platform for intervention. Consumer-grade wearable devices (accelerometers, heart-rate monitors, smart watches) now enable continuous, low-cost estimation of physical activity intensity and volume in adolescents’ naturalistic environments, addressing a key limitation of school-based assessments that capture only a single time-point per academic year. Digital fitness monitoring can also support school-based surveillance by enabling tracking of individual fitness trajectories longitudinally, rather than relying on single cross-sectional measurements that cannot distinguish between adolescents on improving versus declining trajectories. The sensitivity of adolescent fitness to contextual disruption documented in Section 7.3 (the COVID-19 natural experiment) implies that digital tools capable of detecting fitness deterioration in near real-time—and triggering timely support—could have substantial preventive value. Future research should evaluate whether wearable-based longitudinal fitness monitoring in schools can provide the multi-wave trajectory data that current cohort studies largely lack (Section 10).

## 12. Directions for Future Research

Based on the gaps identified above, several priorities emerge for future longitudinal research on HRPF in the 12–16-year age range. First, studies should increasingly incorporate maturity-status indicators (e.g., peak height velocity offset, pubertal staging) alongside chronological age, to disentangle maturational from purely age-related contributions to both fitness change and physical activity decline. Second, greater use of three-or-more-wave designs spanning the full 12–16-year window, rather than two-point baseline–follow-up designs, would allow more precise characterization of trajectory shape (e.g., confirming or refuting the apparent age-15 inflection point identified in cardiorespiratory fitness data). Third, harmonization or at least transparent cross-walking of test batteries (ALPHA, Eurofit, FITNESSGRAM) and tracking statistics (correlation-based, regression-based, categorical) across studies would substantially improve comparability and enable future quantitative (meta-analytic) synthesis specific to this age band, which the present heterogeneous evidence base does not yet support. Fourth, further work is needed on the asymmetry in upward versus downward mobility across fitness tertiles identified in Section 5.2, including testing whether targeted intervention can improve the relatively poor prospects of low-fitness adolescents moving into higher fitness categories—direct intervention trials are needed before tracking-based intervention-timing hypotheses in this review can be considered established. Finally, given the demonstrated sensitivity of adolescent fitness trajectories to short-term contextual disruption (Section 7.3), longitudinal cohorts spanning the 12–16-year window are well placed to evaluate the fitness impact of other environmental and policy changes (e.g., school physical education reforms, digital/sedentary behavior trends) as they arise.

## 13. Conclusions

Longitudinal evidence drawn from cohort and tracking studies indicates that health-related physical fitness continues to develop across the 12–16-year age range but does so unevenly: cardiorespiratory fitness rises through early adolescence before plateauing or declining from around age 15 in a single Icelandic cohort, particularly in boys; muscular strength and power diverge increasingly by sex; flexibility remains comparatively stable; and body composition functions as both an outcome in its own right and a determinant of performance on other fitness tests. Within this same window, fitness rank becomes substantially more stable than it was across the childhood-to-adolescence transition, with the strongest tracking coefficients of all typically observed between mid- and late-adolescent time points— most directly demonstrated in a single longitudinal cohort of adolescent girls. This combination of continuing, sex-divergent development alongside strengthening rank stability positions early-to-mid adolescence as a critical and time-limited opportunity for fitness-promoting intervention—one that, according to the secular trend literature is worth further evaluation given, is increasingly necessary given documented declines in population-level youth fitness across many (though not all) national contexts over recent decades. Future longitudinal research that incorporates maturity status, multiple measurement waves, and harmonized assessment and tracking methodology and direct intervention trials will be needed to refine these conclusions and to better identify the specific sub-periods and subgroups for whom intervention would be most effective. Given the heterogeneity of test batteries and tracking statistics, the frequent overlap of sampled age ranges with the 12–16-year window rather than exact correspondence to it, and the other methodological limitations summarized in Section 10, the age-specific and intervention-timing conclusions drawn above should be regarded as provisional hypotheses generated by the reviewed evidence rather than as established, evaluated recommendations.

## Figures and Tables

**Table 1:** Study characteristics, follow-up duration or assessment window, and baseline assessment battery of key longitudinal, tracking, and secular-trend studies included in this structured narrative review.

Ref.	Study Design	Sample	Follow-Up Window	Theme
Group A: Developmental change and tracking				
[8]	Longitudinal cohort	Adolescent girls; baseline age 15 y	3-year follow-up (15 → 17)	Tracking
[11]	Longitudinal cohort	Adolescent girls; ages 15–17	3-year follow-up	Body composition/change
[4]	Longitudinal cohort	Boys and girls; not specified; ages 7–17 (Iceland)	10-year follow-up; multiple waves	CRF trajectory
[12]	Systematic review and meta-analysis	Children and adolescents across multiple cohorts	Childhood/adolescence to adulthood (variable follow-up duration)	Tracking
[16]	Longitudinal cohort (5 waves)	Mixed sex; ages 6–18	12-year follow-up; 5 time points	Tracking/mobility
Group B: Sex differences and biological maturation				
[5]	Longitudinal cohort	Boys and girls; school-age through mid-adolescence	Multi-year follow-up; timing not specified	Maturation/PA
[17]	Auxological longitudinal cohort	Boys and girls; ages ~10–18	serial height measures	Maturation methods
[18]	Longitudinal cohort (somatic maturity model)	Boys and girls; broad age range	Serial height measures over adolescence	Maturation methods
[9]	Auxological longitudinal cohort	Boys and girls (North American reference sample)	Repeated anthropometry	Growth standards
[10]	Scoping review	Young athletes; broad age range	Variable (scoping review across multiple studies)	PHV/strength/power
Group C: Secular trends and population surveillance				
[13]	Pooled cross-sectional trend analysis	Children and adolescents; 19 countries, ~1981–2014	Repeated cross-sections over 33 years	Secular trend (CRF)
[14]	Systematic review of epidemiological studies	Children and adolescents; 24 studies; *n* > 860,000; 16 countries	Studies published post-2006; variable assessment periods	Secular trend (multi-component)
[15]	Systematic review	Children and adolescents; 6–18 y; multiple countries	Studies spanning 1972–2015	Secular trend (multi-component)
[19]	Repeated national cross-sectional surveys	Chinese adolescents; ages 13–18; 8 successive surveys	1985–2019 (34 years; ~5-year intervals)	Secular trend (China)
[20]	Repeated national cross-sectional surveys	Rural Chinese children/adolescents; 7–18 y	1985–2019 (5-year intervals)	Secular trend (rural China)
[21]	Repeated cross-sectional surveillance	Brazilian children and adolescents; *n* > 65,000; 2005–2022	Repeated cross-sections; 17 years	Secular trend (Brazil)
[22]	Repeated cross-sectional surveillance	Croatian children/adolescents; 11–14 y	1999–2014 (repeated cross-sections)	Secular trend (Croatia)
[23]	National monitoring study protocol	Slovenian children and adolescents; all school-age groups	5 decades; periodic cross-sections	Secular trend (Slovenia)
[24]	Longitudinal cohort; natural experiment (COVID-19)	Portuguese adolescents; grades 5–12; mixed sex	1-year follow-up; 3 time points (pre-lockdown, post-lockdown, 2 months after return)	Contextual disruption
[25]	Systematic review and meta-analyses	European children and adolescents; multiple cohorts	Pre- vs. post-COVID-19 pandemic (2020 onward)	Secular trend (COVID era)
Group D: Health outcomes				
[1]	Narrative review	Children and adolescents; broad age range	Cross-sectional and longitudinal studies (variable)	Health outcomes
[2]	Systematic review and meta-analysis	Children and adolescents; variable age ranges	Longitudinal studies (variable follow-up)	Health outcomes (CRF)
[3]	Systematic review and meta-analysis	Children and adolescents	Longitudinal studies (variable follow-up)	Health outcomes (muscular)
[26]	Systematic review of longitudinal studies	Children and adolescents	Longitudinal (variable follow-up durations)	Health outcomes (CVD risk)
[27]	Longitudinal cohort	Boys and girls; childhood to adulthood	Childhood to adulthood follow-up	Health outcomes (metabolic)
[28]	Systematic review and meta-analysis	Children and adolescents	Cross-sectional and longitudinal (pooled)	Health outcomes (mental)
[29]	Systematic review	Children and adolescents	Cross-sectional and longitudinal (pooled)	Health outcomes (muscular/cardio metabolic)

CRF: cardiorespiratory fitness; CVD: cardiovascular disease; PA: physical activity; PHV: peak height velocity.

**Table 2:** Comparison of the ALPHA, Eurofit, and FITNESSGRAM health-related fitness test batteries.

HRPF Component	ALPHA	Eurofit	FITNESSGRAM
Cardiorespiratory fitness	20 m shuttle run	20 m multistage shuttle run	PACER shuttle run (or 1-mile run)
Muscular strength/power	Handgrip strength; standing broad jump	Handgrip strength; standing broad jump; bent-arm hang	Push-up test; curl-up test
Flexibility	Back-saver sit-and-reach	Sit-and-reach	Back-saver sit-and-reach; shoulder stretch
Body composition	BMI; skinfolds; waist circumference	BMI; skinfolds	BMI; skinfolds or bioelectrical impedance

BMI: body mass index; PACER: Progressive Aerobic Cardiovascular Endurance Run.

**Table 3:** Summary of developmental trajectories for major HRPF components across approximately 12–16 years, by sex.

Component	Boys (~12–16 y)	Girls (~12–16 y)	Source(s)
Cardiorespiratory fitness (absolute)	Rises to 15, then plateaus	Rises to 15, then plateaus	[4]
Cardiorespiratory fitness (relative to lean mass)	Plateaus then declines 15–17	Plateaus from 15 (less decline)	[4]
Muscular strength/power (absolute)	Steep, accelerating gains	Slower, gradual gains	[16,19]
Flexibility	Relatively stable	Relatively stable	[8,12]
Body composition (fat mass)	Stable/modest increase	Tends to increase	[11]

**Table 4:** Tracking statistics for HRPF components, by age span and source.

HRPF Component	Age Span	Statistic	Reported Value/Pattern	Source
Lower-limb explosive power	15 → 17 y	Tracking coefficient (β)	0.98 (highest of all components measured)	[8]
Flexibility	15 → 17 y	Tracking coefficient (β)	0.89	[8]
Body composition	15 → 17 y	Tracking coefficient (β)	0.88	[8]
Speed endurance	15 → 17 y	Tracking coefficient (β)	0.86	[8]
Aerobic endurance	15 → 17 y	Tracking coefficient (β)	0.75	[8]
Muscular endurance (lower limb)	15 → 17 y	Tracking coefficient (β)	0.65	[8]
Muscular endurance (trunk)	15 → 17 y	Tracking coefficient (β)	0.51 (lowest of all components measured)	[8]
Cardiorespiratory fitness (category)	15 → 17 y	Odds ratio (same category)	Highest OR of all age comparisons tested	[4]
Cardiorespiratory fitness (category)	9 → 15 y; 9 → 17 y	Odds ratio (same category)	Statistically significant, but lower than 15 → 17 y	[4]
Cardiorespiratory fitness (category)	7 → 15 y; 7 → 17 y	Odds ratio (same category)	Not statistically significant	[4]
CRF, muscular strength, muscular endurance	Childhood/adolescence → adulthood	Pooled tracking correlation	Moderate; slightly higher tracked from adolescence than childhood	[12]
Flexibility	Childhood/adolescence → adulthood	Pooled tracking correlation	Highest of all components in this meta-analysis	[12]
Composite fitness (multi-test)	Lowest tertile at baseline, ages 6–18	Probability of reaching top tertile later	Markedly low; middle tertile has substantially better odds	[16]

CRF: cardiorespiratory fitness; OR: odds ratio; β: standardized tracking (stability) coefficient from regression-based models.

**Table 5:** Secular trends in health-related physical fitness in children and adolescents, by region and study period.

Region/Country	Study Period	Sample	Age Range (y)	Principal Trend
16 countries (pooled)	Studies to 2006–present	*n* > 860,000	6–18	Majority decline in endurance, strength, and flexibility; speed/coordination inconsistent [14]
Multinational (pooled update)	1972–2015	Multiple cohorts	6–18	~10% decline 1975–2000, most pronounced for endurance/flexibility; decline larger in adolescents than children; CRF decline now flattening [15]
19 high-/upper-middle-income countries	1981–2014	Multiple national cohorts	9–17 (approx.)	Sample-weighted 7.3% decline in cardiorespiratory fitness [13]
Slovenia	5 decades (ACDSi programme)	National decennial surveillance	Schoolchildren/adolescents	CRF decline largest, now stabilising (~2012+); strength modestly improving; power still declining; speed improving in subgroups [23]
Croatia	1999–2014	National surveillance	11–14	General decline in health-related fitness, consistent with Slovenian/pooled data [22]
China (national)	1985–2019	8 successive national surveys	13–18	Height, weight, and BMI rose steadily over 34 years [19]
China (rural)	1985–2019	National surveillance	7–18	Fitness rose 1985–2000, fell 2000–2010, mixed component-specific rebounds 2010–2019 [20]
Brazil	2005–2022	Repeated cross-section, *n* > 65,000	Children/adolescents	Fitness declining for the majority; decline asymmetric and accelerating in recent years; “fit minority” stable [21]
Europe (pooled)	Pre- vs. post-COVID-19	Systematic review/meta-analyses	Children/adolescents	HRPF weakened across the pandemic period relative to pre-pandemic trajectories [25]

BMI: body mass index; CRF: cardiorespiratory fitness.

**Table 6:** Recurring methodological limitations in longitudinal HRPF research, their interpretive consequences, and recommended mitigations.

Limitation	Consequence	Mitigation
Test battery/outcome heterogeneity	Tracking/change estimates not directly comparable	Report both absolute and relative values; standardized units [4,16]
Variability in tracking statistics	Statistics not numerically interchangeable	Favor narrative synthesis over direct numerical; report the statistic type [4,8,12]
Short follow-up/few waves	Cannot distinguish trajectory shape	Prioritize ≥3-wave designs [4,8,16]
Attrition	Bias toward advantaged subgroups	Report attrition by baseline fitness/SES [8]
Age–maturation confounding	Misattribution of maturation vs. age effects	Incorporate maturity-offset methods [6,40]
Secular confounding	Same age band, different eras differ	Interpret against contemporaneous secular data [14,15,20,21]

HRPF: health-related physical fitness; SES: socioeconomic status.

## Data Availability

No new data were created or analyzed in this study. Data sharing is not applicable to this article.
